# Filth flies are transport hosts of Cryptosporidium parvum.

**DOI:** 10.3201/eid0505.990520

**Published:** 1999

**Authors:** T K Graczyk, R Fayer, M R Cranfield, B Mhangami-Ruwende, R Knight, J M Trout, H Bixler


**Acknowledgment**

We thank Jane E. Raulston for review of the manuscript and for useful suggestions.

**Jerome L. Sullivan* and Eugene D. Weinberg†**
*University of Florida College of Medicine, Gainesville, Florida, USA; and †Indiana University, Bloomington, Indiana, USA

### References

1. Saikku P, Mattila K, Nieminen MS, Huttunen JL, Leinonen M, Ekman M-R, et al. Serological evidence of an association of a novel *Chlamydia*, TWAR, with chronic coronary heart disease and acute myocardial infarction. Lancet 1988;ii:983-5.
2. Saikku P. The epidemiology and significance of *Chlamydia pneumoniae*. J Infect 1992;25 Suppl I:27-34.
3. Saikku P, Leinonen M, Tenkanen L, Linnanmaki E, Ekman MR, Manninen V, et al. Chronic *Chlamydia pneumoniae* infection as a risk factor for coronary heart disease in the Helsinki Heart Study. Ann Intern Med 1992;15;116:273-8.
4. Sullivan JL. Iron and the sex difference in heart disease risk. Lancet 1981;1:1293-4.
5. Sullivan JL. Iron versus cholesterol—perspectives on the iron and heart disease debate. J Clin Epidemiol 1996;49:1345-52.
6. Salonen JT, Nyyssonen K, Korpela H, Tuomilehto J, Seppanen R, Salonen R. High stored iron levels are associated with excess risk of myocardial infarction in eastern Finnish men. Circulation 1992;86:803-11.
7. Weinberg ED. Patho-ecologic implications of microbial acquisition of host iron. Reviews in Medical Microbiology 1998;9:171-8.
8. Freidank HM, Billing H. Influence of iron restriction on the growth of *Chlamydia pneumoniae* TWAR and *Chlamydia trachomatis*. Clinical Microbiology and Infection 1997;3 Suppl 2:193.
9. Thong PSP, Selley M, Watt F. Elemental changes in atherosclerotic lesions using nuclear microscopy. Cell Mol Biol 1996;42:103-10.
10. Lakka TA, Nyyssönen K, Salonen JT. Higher levels of conditioning leisure time physical activity are associated with reduced levels of stored iron in Finnish men. Am J Epidemiol 1994;140:148-60.

### Filth Flies Are Transport Hosts of *Cryptosporidium parvum*

**To the Editor:** Infection with *Cryptosporidium parvum,* a zoonotic and anthroponotic coccidian parasite (1), may be fatal for persons with impaired immune systems (2), for whom a low number of oocysts can initiate life-threatening diarrhea (1). Insects such as promiscuous-landing synanthropic flies (i.e., coprophilic filth flies) are recognized transport hosts for a variety of parasites (3-5), but not for *C. parvum*. We assessed the role of synanthropic flies in the mechanical transmission of *C. parvum* oocysts.

Bovine diarrheic feces (20-ml specimens) containing 2.0 x $10^5$ oocysts/ml were placed in petri dishes in each of five 4-liter paper cages with approximately 250 pupae of laboratory-reared house flies (*Musca domestica* F58WTZ strain). Three days after the flies emerged, fecal specimens were collected on glass microscope slides placed in each cage. Thirty flies aspirated from each cage on days 3, 5, 7, 9, and 11 after emergence were eluted, and the eluants were processed by the cellulose acetate membrane (CAM)-filter dissolution method (6). Digestive tracts dissected from randomly selected flies and the glass slides with fly excreta were examined by immunofluorescent antibody (IFA) (7), and *C. parvum* oocysts were counted (8). Maggots of *M. domestica* were reared in fly larvae medium (PMI FEEDS, Inc., St. Louis, MO) contaminated with calf diarrheic feces (50 ml) containing 2.0 x $10^5$ *C. parvum* oocysts/ml. Resulting pupae were eluted, the eluants were processed by the CAM-filter dissolution method (6), and *C. parvum* oocysts were identified by IFA (7) and counted (8). Diarrheic fecal specimens from a *C. parvum*-uninfected calf were used as negative controls in similar experiments. Randomly selected samples containing fly-derived *C. parvum* oocysts were processed with acid-fast stain (AFS) (8) to check for normal cellular morphologic features.

Ten Victor-type flying-insect traps (Woodstream, Lititz, PA) were baited with rotten fish and placed inside a barn (approximately 880 $m^2$) in which a male Holstein calf infected with *C. parvum* (AUCP-1 strain) was housed. The traps were emptied weekly, the flies were counted and identified (5,9), and the inside surfaces of the traps (containing fly excreta), along with the flies, were eluted with 200 ml of eluting fluid (6). The eluting fluid was filtered through a CAM (Millipore, Bedford, MA) (6,8), which was then processed (6), and *C. parvum* oocysts were identified by IFA (7) and counted (8).

The mean number of *C. parvum* oocysts per droplet of *M. domestica* was 4 to 20 (mean 7.0 $\pm$ 3.2), and the number of droplets increased over time. All flies harbored *C. parvum* oocysts on their external surfaces. On average, 14.0 + 6.8 fly excreta were counted per 1.0 $cm^2$ of glass slide. From 1 to 8 *C. parvum* oocysts were detected in digestive tracts of flies exposed to feces with oocysts. *C. parvum* oocysts were also numerous on maggot and pupa surfaces; approximately 150 and 320 oocysts were recovered per maggot and pupa, respectively.

Wild-caught flies belonged to the families *Calliphoridae* (96% of total flies), *Sarcophagidae* (2%), and *Muscidae* (2%). An average of eight flies was caught per trap, and more than 90% of flies harbored *C. parvum* oocysts. The number of trap-recovered *C. parvum* oocysts per fly was 2 to 246 (mean 73 oocysts per fly).

Synanthropic flies that breed in or come in contact with a fecal substrate contaminated with *C. parvum* oocysts can harbor these oocysts both externally and internally and will mechanically deposit them on other surfaces. Therefore, synanthropic flies can serve as mechanical vectors for *C. parvum* oocysts and under poor sanitary conditions could be involved in the transmission of human and animal cryptosporidiosis. The biology and ecology of synanthropic flies indicate that their potential for mechanical transmission of *C. parvum* oocysts can be high. The morphologic and AFS and IFA staining characteristics of *C. parvum* oocysts recovered from the exoskeletons of flies and identified in their fecal spots suggest that oocysts are still viable.

**Thaddeus K. Graczyk,*† Ronald Fayer,‡ Michael R. Cranfield,*† Barbara Mhangami-Ruwende,* Ronald Knight,* James M. Trout,§ and Heather Bixler§**

*Johns Hopkins University, Baltimore, Maryland, USA; †The Baltimore Zoo, Druid Hill Park, Baltimore, Maryland, USA; ‡U.S. Department of Agriculture, Beltsville, Maryland, USA; and §University of Pennsylvania, School of Veterinary Medicine, Philadelphia, Pennsylvania, USA

### References

1. Fayer R, Speer CA, Dubey JP. The general biology of *Cryptosporidium*. *Cryptosporidium* and cryptosporidiosis. In: Fayer R, editor. Boca Raton (FL): CRC Press; 1997. 1-42.
2. Graczyk TK, Fayer R, Cranfield MR. Zoonotic potential of cross-transmission of *Cryptosporidium parvum*: implications for waterborne cryptosporidiosis. Parasitol Today 1996;13:348-51.
3. Wallace GD. Experimental transmission of *Toxoplasma gondii* by filth-flies. Am J Trop Med Hyg 1971;20:411-3.
4. Hedges SA. Flies, gnats and midges. In: Malis A, editor. Handbook of pest control. Cleveland (OH): Franzak & Foster Co.; 1990. p. 621-84.
5. Greenberg B. Flies and diseases, biology and disease transmission. Princeton (NJ): Princeton University Press; 1973. p. 221.
6. Graczyk TK, Cranfield MR, Fayer R. Recovery of waterborne oocysts of *Cryptosporidium parvum* from water samples by the membrane-filter dissolution method. Parasitol Res 1997;83:121-5.
7. Graczyk TK, Cranfield MR, Fayer R. Evaluation of commercial enzyme immunoassay (EIA) and immunofluorescent antibody (IFA) test kits for detection of *Cryptosporidium* oocysts of species other than *Cryptosporidium parvum*. Am J Trop Med Hyg 1996;54:274-9.
8. Graczyk TK, Fayer R, Cranfield MR, Owens R. *Cryptosporidium parvum* oocysts recovered from water by the membrane filter dissolution method retain their infectivity. J Parasitol 1997;83:111-4.
9. Borror DJ, DeLong DM, Triplehorn CA. An introduction to the study of insects. Philadelphia (PA): Saunders College Publishing; 1981. p. 827.

### The Cost-Effectiveness of Vaccinating against Lyme Disease

**To the Editor:** The recent article by Meltzer and colleagues (1) is an important contribution to a pertinent public health issue: who should receive the newly licensed Lyme disease vaccine. Answering this question is a daunting task, given the scarcity of valid data. Estimates of the spectrum and prevalence of the long-term sequelae of Lyme disease remain controversial (2-4). In generating their cost-effectiveness model, Meltzer et al. examined the cost savings involved in preventing three categories of classic organ-specific Lyme disease sequelae (cardiovascular, neurologic, and arthritic); however, they did not take into account the potential cost savings from preventing cases of a generalized symptom complex known as post-Lyme syndrome, which includes persisting myalgia, arthralgia, headache, fatigue, and neurocognitive deficits. These generalized sequelae, which are recognized by the National Institutes of Health as late sequelae of Lyme disease, have been found to persist for years after antibiotic therapy (5,6). Two population-based retrospective cohort studies (7,8) among Lyme disease patients whose illness was diagnosed in the mid-1980s determined that one third to half had clinically corroborated post-Lyme syndrome symptoms years after the initial onset of disease. Although these studies were conducted 15 years ago, when optimal antibiotic regimen guidelines were still evolving, the estimated cost of averting these often-disabling nonorgan-specific symptoms should also be taken into account in estimated

